# DIR-based models to predict weekly anatomical changes in head and neck cancer proton therapy

**DOI:** 10.1088/1361-6560/ac5fe2

**Published:** 2022-04-15

**Authors:** Ying Zhang, Stacey McGowan Holloway, Megan Zoë Wilson, Jailan Alshaikhi, Wenyong Tan, Gary Royle, Esther Bär

**Affiliations:** 1 Department of Medical Physics and Biomedical Engineering, University College London, Gower Street, London WC1E 6BT, United Kingdom; 2 Saudi Proton Therapy Center, King Fahad Medical City, Riyadh, Saudi Arabia; 3 Department of Oncology, Shenzhen Hospital of Southern Medical University Shenzhen 518101, People's Republic of China; 4 University College London Hospitals NHS Foundation Trust, Radiotherapy Physics, 250 Euston Road, London NW1 2PG, United Kingdom; 5 CRUK RadNet Glasgow, University of Glasgow, Beatson West of Scotland Cancer Centre, Radiotherapy Physics, NHS Greater Glasgow and Clyde, Glasgow, United Kingdom

**Keywords:** anatomical model, uncertainty evaluation, proton therapy

## Abstract

*Objective*. We proposed two anatomical models for head and neck patients to predict anatomical changes during the course of radiotherapy. *Approach*. Deformable image registration was used to build two anatomical models: (1) the average model (AM) simulated systematic progressive changes across the patient cohort; (2) the refined individual model (RIM) used a patient’s CT images acquired during treatment to update the prediction for each individual patient. Planning CTs and weekly CTs were used from 20 nasopharynx patients. This dataset included 15 training patients and 5 test patients. For each test patient, a spot scanning proton plan was created. Models were evaluated using CT number differences, contours, proton spot location deviations and dose distributions. *Main results*. If no model was used, the CT number difference between the planning CT and the repeat CT at week 6 of treatment was on average 128.9 Hounsfield Units (HU) over the test population. This can be reduced to 115.5 HU using the AM, and to 110.5 HU using the RIM_3_ (RIM, updated at week (3). When the predicted contours from the models were used, the average mean surface distance of parotid glands can be reduced from 1.98 (no model) to 1.16 mm (AM) and 1.19 mm (RIM_3_) at week 6. Using the proton spot range, the average anatomical uncertainty over the test population reduced from 4.47 ± 1.23 (no model) to 2.41 ± 1.12 mm (AM), and 1.89 ± 0.96 mm (RIM_3_). Based on the gamma analysis, the average gamma index over the test patients was improved from 93.87 ± 2.48 % (no model) to 96.16 ± 1.84% (RIM_3_) at week 6. *Significance*. The AM and the RIM both demonstrated the ability to predict anatomical changes during the treatment. The RIM can gradually refine the prediction of anatomical changes based on the AM. The proton beam spots provided an accurate and effective way for uncertainty evaluation.

## 1. Introduction

Proton therapy has demonstrated great potential in limiting the dose to normal tissue adjacent to the target region for head and neck (H&N) cancer patients (Mitin and Zietman 2014, Leeman *et al* 2017). However, the sharp distal fall-off of the Bragg peak makes the dose distribution sensitive to geometrical variations (Lomax 2008, McGowan *et al* 2013), which are especially common in the treatment of H&N cancer. Tan *et al* (2013) showed that the tumour volume of 20 nasopharynx cancer patients on average reduced by 36.5% during the treatment ranging from 20% to 60%. Additionally, organs at risk (OARs) lose cells under irradiation leading to complications such as dysphagia (swallowing difficulties) and dysgeusia (taste changes), often accompanied with weight loss and the shrinkage of the patient’s outline (Yan *et al* 2013). What follows are changes in the positions of target and OARs. Bhide *et al* (2010) showed the parotid volume of 20 H&N patients decreased with a reduction rate between 21.3% and 42%, and an average of 2.3 mm medial shift occurred by the fourth week of treatment.

With these anatomical changes during the course of treatment, dose degradation is unavoidable (Kraan *et al* 2013, Wu *et al* 2017, Heukelom *et al* 2019). Kraan *et al* (2013) showed in 10 oropharyngeal cancer patients that anatomical changes lead to an average of 2% and 2.2% reduction in the D98% of high risk clinical target volume (CTV) and low risk CTV, respectively, and the maximum increase in brainstem dose can reach 9.2 Gy. Wu *et al* (2017) showed in 10 oropharyngeal patients that mean doses to the CTV were reduced up to 7%, while an increase was shown in the right parotid with a range from 5% to 8%.

The dose degradation from anatomical changes is currently addressed using adaptive radiotherapy in proton centres. However, while plans are adapted, patients either continue treatment with an existing sub-optimal plan or face interruptions to treatment. To prepare offline adaptation in advance, anatomical modelling aims to provide accurate deformations that include individual progressive changes. An individual model is built based on individual patient images acquired during the first *F* fractions and predicts the anatomical changes of the following fractions for that particular patient (Van Kranen *et al* 2013, Chetvertkov *et al* 2016). Ideally, an anatomical model that can provide accurate predictions before treatment would be most beneficial to clinical practice. However, the effect of inter-fraction variations and the influence of acute toxicity on patients during the treatment also affect the anatomy. An alternative is to develop a model capturing the systematic progressive changes based on population data. Each patient’s model could then be refined as patient-specific data are acquired over the course of treatment.

In this study, we developed two anatomical models based on deformable image registration (DIR). The objectives of this work are: (1) to develop an average model (AM) based on population data to predict the weekly systematic progressive changes before treatment. (2) To refine the prediction by adding the patient-specific progressive information from the data acquired during the course of treatment, known as the refined individual model (RIM). (3) To evaluate the models using Hounsfield Units (HU) differences, contours, proton spot location deviations and intensity-modulated proton therapy (IMPT) dose distributions.

## 2. Method and material

### 2.1. Patient data

Twenty patients with treatment-naïve, locally advanced nasopharyngeal carcinoma were recruited retrospectively. Each patient underwent a planning CT (pCT) and six weekly verification CT (rCT_*t*_), where *t* (*t* = 0, 1, 2, 3,…) represents the week of CT scanning. The image acquisition details can be found in Appendix A. Contours in the pCT and rCTs were manually delineated by an oncologist. Five of the 20 patients were held separate as a test set, and the model was built using the remaining 15 patients.

For all 5 test patients, an original (nominal) IMPT treatment plan with three beam fields (60°, 180°, 300°) was generated using the Eclipse version 16.1.0 (Varian Medical Systems, Palo Alto, CA). All plans generated throughout this study were robustly optimised with ±3 mm setup and ±3.5% range uncertainty for CTVs and critical OARs. A relative biological effectiveness of 1.1 for proton beams was used. The dosimetric goals for all plans in this study are summarised in table 1. A plan was deemed acceptable if the goals set for the CTV and serial organs were fulfilled in the nominal scenario (the error-free distribution) as well as all 12 dose distributions (3 mm orthogonal shifts combined with the ±3.5% range error) in a robust evaluation. More clinical characteristics of the patients can be found in the paper of Tan *et al* (2013, 2016).

**Table 1.** Dosimetric goals of the treatment plans created in this study.

Structure	Goal under uncertainty
High-risk-CTV	D_95_(The minimum dose to 95% of target volume) >95% of prescription dose (72.6 Gy, 33 fractions)
Low-risk-CTV	D_95_ > 95% of prescription dose (63 Gy, 33 fractions)
CTV	D_2_ (The minimum dose to the hottest 2% volume ) <107% of prescription dose
Spinal cord	${{\mathrm{D}}}_{\max }$ (The maximum dose in the volume) <45 Gy
Brainstem	${{\mathrm{D}}}_{\max }$ <55 Gy
Chiasm	${{\mathrm{D}}}_{\max }$ <55 Gy
Structure	Goal in nominal
Parotid glands	D_mean_ (The mean dose in the volume) <26 Gy
Oral cavity	D_mean_ <40 Gy
Larynx	D_mean_ <40 Gy
Proton planning information: MFO planning; spot spacing size: 5mm; energy range: 70–250 MeV; range shifter: 5 cm	
dose calculation algorithm: Piencel beam scanning (PBS); optimisation algorithm: Nonlinear universal Proton Optimizer.	

### 2.2. Anatomical models

This section describes the DIR-based models that were built at each weekly time point considering the time dependence of the progressive changes. The AM used the average deformation of each week for prediction. The RIM further refined the prediction of the AM by adding the deformation difference at the early treatment between the actual deformation acquired during the treatment and the average deformation. This deformation difference represents the progressive difference between individuals.

To help the reading and understanding of this paper, the symbols and abbreviations used in this paper are listed in table 2.

**Table 2.** A glossary of defined variables and acronyms.

Full name	Abbreviation
Planning CT	pCT
Weekly verification CT at treatment week t	rCT_*t*_
Deformed rCT that was similar to its respective planning CT	dCT_*t*_
Deformation vector field	DVF ***ϕ***
Stationary velocity field	SVF ***v*** (${\boldsymbol{\phi }}=\exp ({\boldsymbol{v}}$)
The SVF that registered rCT_*t*_ to pCT	***v***_*p*→*t*_
The SVF that registered pCT to rCT_*t*_	***v***_*t*→*p*_
The SVF between pCT and the atlas	***R***_*a*→*p*_
***v***_*p*→*t*_ in the atlas space	***v***_*a*,*p*→*t*_
The number of patients used in the models	*N*_*p*_
Patient index	*pi*
Average model	AM
The SVF of the AM used for the prediction at week t	${{\boldsymbol{v}}}_{t\to p}^{\mathrm{AM}}$
The predicted CT of the AM at treatment week *t*	CT${}_{t}^{\mathrm{AM}}$
Total treatment week	n
Refined individual model	RIM
The SVF of the individual random deformation at week *t*	${{\boldsymbol{v}}}_{t+i\to p}^{{ind}}$
The SVF of the RIM used for the prediction at week *t*	${{\boldsymbol{v}}}_{t+i\to p}^{\mathrm{RIM}}$
The predicted CT of the AM at treatment week *t*	CT${}_{t}^{\mathrm{RIM}}$
Average absolute Hounsfield Units difference	AAHUD
Water equivalent path length	WEPL
The deviation of a spot position ***r*** on the beam path	*σ*(***r***)
The normalized spot weight	*w*_*r*_
Weighted spot location deviation	WSLD
Confidence interval	CI

#### 2.2.1. Deformable image registration

Deformation vector fields (DVFs) from DIR are often used to build anatomical models (Van Kranen *et al* 2013, Chetvertkov *et al* 2016, Yu *et al* 2016). The deformation fields find the optimal transformation to achieve the greatest similarity between two images. This transformation can be physically realised as a DVF *ϕ*, which encodes the three-dimensional motion of the voxels in the reference image. In this work, we used DIR to register weekly CTs of patient data sets to their pCT.

To ensure that the inter-fraction DVFs of patients were in the same space and had the same resolution, we projected the DVFs into the atlas space. The atlas was obtained from a group-wise registration which spatially normalised a cohort of patients in a common space in order to statistically quantify global or local differences between groups of subjects.^6^ 
^6^

https://cmiclab.cs.ucl.ac.uk/mmodat/niftyreg. The procedure of generating an atlas is illustrated in appendix B. In the procedure of the projection, pCT was the reference geometry, each rCT_*t*_ was deformed to its respective pCT to produce transformation *ϕ*_*p*→*t*_, where *p* stands for pCT and dCT_*t*_ is the deformed rCT_*t*_ using the weekly transformation field of *ϕ*_*p*→*t*_. After that, each patients pCT was registered to the atlas to produce *ϕ*_*a*→*p*_, where *a* stands for atlas. *ϕ*_*a*→*p*_ transformed the inter-patient velocity fields *ϕ*_*p*→*t*_ into the atlas space using

\begin{eqnarray*}{\phi }_{a,p\to t}={\phi }_{a\to p}^{-1}\,\circ \,{\phi }_{p\to t}\,\circ \,{\phi }_{a\to p}.\end{eqnarray*}

Because the prediction direction is from pCT to rCT_*t*_, the transformation needs inverting. To easily calculate the inverse transformation, the exponential map was given by Lie group (Hall 2003). DVFs *ϕ* can be expressed as

\begin{eqnarray*}\phi =\exp ({\boldsymbol{v}}),\end{eqnarray*}

where ***v*** is the stationary velocity field (SVF) of the diffeomorphic image registration (Avants *et al* 2008, Ehrhardt *et al* 2010) used to identify anatomical changes in this project.

The inverse transformation *ϕ* ^−1^ can now be calculated as

\begin{eqnarray*}\phi =\exp ({\boldsymbol{v}})\hspace{4mm}\Rightarrow \hspace{4mm}{\phi }^{-1}(x)=\exp (-{\boldsymbol{v}}).\end{eqnarray*}

We used ***v*** instead of *ϕ* to capture the inter-fraction transformation, and to distinguish from the inter-fraction ***v***, we used ***R***_*a*→*p*_ to present the SVF from pCT to altas CT. The projection equation (1) changed into:

\begin{eqnarray*}{{\boldsymbol{v}}}_{a,p\to t}={{\boldsymbol{R}}}_{a\to p}^{-1}\,\circ \,{{\boldsymbol{v}}}_{p\to t}\,\circ \,{{\boldsymbol{R}}}_{a\to p}.\end{eqnarray*}

To be noted here, this equation is the result of an approximation.

The diffeomorphic image registration is a B-splines based method implemented in NiftyReg ^6^(Modat *et al* 2010, 2012). It is invertible, differentiable and whose inverse is also differentiable (Avants *et al* 2008, Modat *et al* 2010, 2012), preserving the underlying topology. NiftyReg is an open-source DIR tool available as part of the NifTK project, developed by computer scientists at Centre for Image Computing (CMIC) within University College London (UCL).

#### 2.2.2. Average model

The first model implemented here was the AM. The weekly SVFs between pCT and the rCT_*t*_ of the training data in the atlas were used as input. The produced predicted CTs presented systematic progressive changes during the course of treatment. The procedure was divided into three steps and repeated for each treatment week.

(i) The SVF for week *t* in the atlas space was calculated as the expectation value *E* of the deformation ***v***_*a*, *p*→*t*_ of the training dataset
  \begin{eqnarray*}{{\boldsymbol{v}}}_{a,p\to t}^{\ \mathrm{AM}}={\bf{E}}({{\boldsymbol{v}}}_{a,p\to t})=\displaystyle \frac{1}{{N}_{p}}\sum _{{pi}}{{\boldsymbol{v}}}_{a,p\to t}^{{pi}},\end{eqnarray*}
  where *N*_*p*_ is the number of patients used in this model and *pi* is the patient index.
(ii) The deformation ${{\boldsymbol{v}}}_{a\to t}^{{AM}}$ was transformed into the space of an individual patient using
  \begin{eqnarray*}{{\boldsymbol{v}}}_{p\to t}^{\mathrm{AM}}={{\boldsymbol{R}}}_{a\to p}\,\circ \,{{\boldsymbol{v}}}_{a,p\to t}^{{AM}}\,\circ \,{{\boldsymbol{R}}}_{a\to p}^{-1}.\end{eqnarray*}
(iii) The predicted patient-specific deformation ${{\boldsymbol{v}}}_{t\to p}^{{AM}}$ was used for warping the pCT to generate the predicted anatomy. In order to warp the pCT, the transformation must be directed from the predicted anatomy to the pCT. This can be simply achieved by reversing the SVFs using
  \begin{eqnarray*}{{\boldsymbol{v}}}_{t\to p}^{\mathrm{AM}}=-{{\boldsymbol{v}}}_{p\to t}^{\mathrm{AM}}.\end{eqnarray*}
  The predicted CT of the AM at treatment week *t* (CT${}_{t}^{\mathrm{AM}}$) can be acquired using
  \begin{eqnarray*}{\phi }_{t\to p}^{\mathrm{AM}}=\exp ({{\boldsymbol{v}}}_{t\to p}^{\mathrm{AM}}),\end{eqnarray*}
  \begin{eqnarray*}{\mathrm{CT}}_{t}^{\mathrm{AM}}={\phi }_{t\to p}^{\mathrm{AM}}(\mathrm{pCT}).\end{eqnarray*}

The AM only considered systematic deformations. The random deformations (progressive variation between patients) can be included by adding individual random deformations using newly acquired weekly CTs of the individual patient during the treatment to gradually refine the prediction of the following weeks, leading to the RIM, described in section 2.2.3.

#### 2.2.3. Refined individual model.

In this section, we proposed the RIM, which is based on the AM but includes the individual random deformations of the specific patient, to further improve the prediction. We assumed that patients share the basic deformation trend during the treatment (AM), e.g. the progressive changes are rapid at the early treatment and then slow down, but with an individual baseline. This baseline as a constant can be corrected in the RIM using the deformation difference between the actual deformation of the patient acquired during the early treatment and the average deformation of the AM and applied to the prediction of the remaining treatment course. Hence, the RIM assumes that, if the shrinkage of the parotid for one patient is visibly more severe compared to the average at fraction i, then the parotid shrinkage of the following fractions is more severe than the average with the same magnitude.

To build the RIM, we applied the AM to the patients pCT first. The procedure to refine the prediction followed the following steps:

(i) We captured the accurate deformation between pCT to rCT_*t*_ during the early treatment, referred to as ***v***_*t*→*p*_. The update started from week 2 because the progressive changes in the first week are less significant (Barker *et al* 2004, Lee *et al* 2008, Stützer *et al* 2017).
(ii) The individual random deformation ${{\boldsymbol{v}}}_{t+i\to p}^{\mathrm{ind}}$ for the remaining fractions can be obtained by
  \begin{eqnarray*}{{\boldsymbol{v}}}_{t+i\to p}^{\mathrm{ind}}={{\boldsymbol{v}}}_{t\to p}-{{\boldsymbol{v}}}_{t\to p}^{\mathrm{AM}}\hspace{2mm},i=1...(n-t),\end{eqnarray*}
  where *n* is the total number of treatment weeks.
(iii) The deformation field ${{\boldsymbol{v}}}_{t+i\to p}^{\mathrm{RIM}}$ for the following fractions as predicted by the RIM can be calculated as
  \begin{eqnarray*}{{\boldsymbol{v}}}_{t+i\to p}^{\mathrm{RIM}}={{\boldsymbol{v}}}_{t+i\to p}^{\mathrm{AM}}+{{\boldsymbol{v}}}_{t+i\to p}^{\mathrm{ind}}\hspace{2mm}i=1...(n-t).\end{eqnarray*}

When treatment starts, we can obtain individual data and use the RIM to gradually update the predicted anatomy. In clinical practice, most H&N plan adaptions occur around the 3rd or 4th week of treatment, we picked *t* = 2, 3 as examples. When *t* = 2, the model was referred to as RIM_2_. When *t* = 3, the model was referred to as RIM_3_.

### 2.3. Model evaluation

#### 2.3.1. Model evaluation based on CT numbers

To assess the anatomical models, we calculated the difference images between predicted CTs and corresponding rCTs. The difference images can be quantified using the average absolute Hounsfield Units difference (AAHUD) within a patient outline.

#### 2.3.2. Model evaluation based on contours

The contours in the predicted images are the propagated contours by applying the deformations of the models to the contours in the pCT. The contour differences between predicted contours and manually delineated contours in rCT (gold standard) were quantified using the three-dimensional mean surface distance (MSD) (Brock *et al* 2017) for each week. The contours included in this evaluation were low risk CTV, high risk CTV and parotid glands. These structures commonly change their shape and volume during the treatment.

#### 2.3.3. Model evaluation based on weighted spot location deviation (WSLD)

In previous uncertainty evaluation work Holloway *et al* (2017), Kim *et al* (2017), the water equivalent path length (WEPL) of a beam to specific points or areas was used. Kim *et al* (2017) quantified the anatomical uncertainty by the WEPL changes on the distal edge of tumour volume. However, only one beam direction was used in their paper. Holloway *et al* (2017) evaluated the uncertainty by the WEPL changes in the CTV with different beam angles, but the analysis of WEPL was limited in the target area. In this work, we used an estimation of the spot location within the patient, derived from the treatment plan file and CT image information. We describe our methods to determine the spot locations in appendix C. Because both spot positions and weights (Li *et al* 2015) affect the dose distribution, the WSLD presented in equation (12) was used to evaluate the uncertainty:

\begin{eqnarray*}\mathrm{WSLD}=\sum _{{\boldsymbol{r}}}\sigma ({\boldsymbol{r}})\cdot {w}_{r},\hspace{2mm}\sum {w}_{r}=1,\end{eqnarray*}

where *w*_*r*_ is the normalized spot weight. *σ*(***r***) is the deviation of each spot on the beam path defined in equation (13):

\begin{eqnarray*}\sigma ({\boldsymbol{r}})=| {{\boldsymbol{r}}}_{\mathrm{uncertainty}}-{{\boldsymbol{r}}}_{\mathrm{reference}}| ,\end{eqnarray*}

where ***r*** is a spot position in the CT, ***r***_reference_ is the spot location in the reference frame and ***r***_uncertainty_ is the spot location under uncertainty. Without having to calculate the dose distribution, the WSLD is accurate and effective in describing the consequences of anatomical deformations.

The WSLD was applied to evaluate (1) the influence of DIR on the calculated spot position; (2) the influence of the systematic anatomical progressions on the spot position and (3) residual anatomical uncertainty.

• Influence of DIR on the spot location—The WSLD between dCTs (dCTs should have the same spot locations as the pCT in ideal DIR) and their corresponding pCTs evaluated the influence of DIR on the spot location.
• The systematic progression uncertainty simulated by the AM—The AM captured the systematic progressive changes of a patient cohort. Therefore, the WSLD estimated by the AM showed the consequence of the systematic progressive changes in the training patient cohort. pCT was used as a reference in equation (13).
• The residual anatomical uncertainty from models—The difference between the estimated anatomical uncertainty from models and actual anatomical uncertainty was used to evaluate the accuracy of the models. We referred to it as the residual anatomical uncertainty (ΔWSLD_res_) from anatomical models, see equation (14),
  \begin{eqnarray*}{\mathrm{\Delta }}{\mathrm{WSLD}}_{\mathrm{res}}={\mathrm{WSLD}}_{\mathrm{real}}-{\mathrm{WSLD}}_{\mathrm{model}},\end{eqnarray*}
  where WSLD_real_ is the actual anatomical uncertainty calculated by the WSLD between rCT and pCT, which is also corresponding to the residual anatomical uncertainty of no model. WSLD_model_ is the anatomical uncertainty estimated by a model. The best model should approach a ΔWSLD_res_ of 0.

#### 2.3.4. Model evaluation based on dose distribution

The nominal plan was recalculated on the rCTs and predicted weekly CTs. The gamma index was used to evaluate the dose difference between the dose distribution on rCTs (**D**_ref_) and predicted weekly CTs (**D**_pred_) (Low *et al* 1998). A relatively stringent criterion of 2 mm/2% and the acceptable passing rate of 95% were used in this study because they are the paired parameters generally used (Park *et al* 2018, Yu *et al* 2019).

## 3. Results

### 3.1. Deformable image registration evaluation

In this section, we evaluated the DIR algorithm based on contours, the influence on the spot location and gamma index.

**Figure 1.**
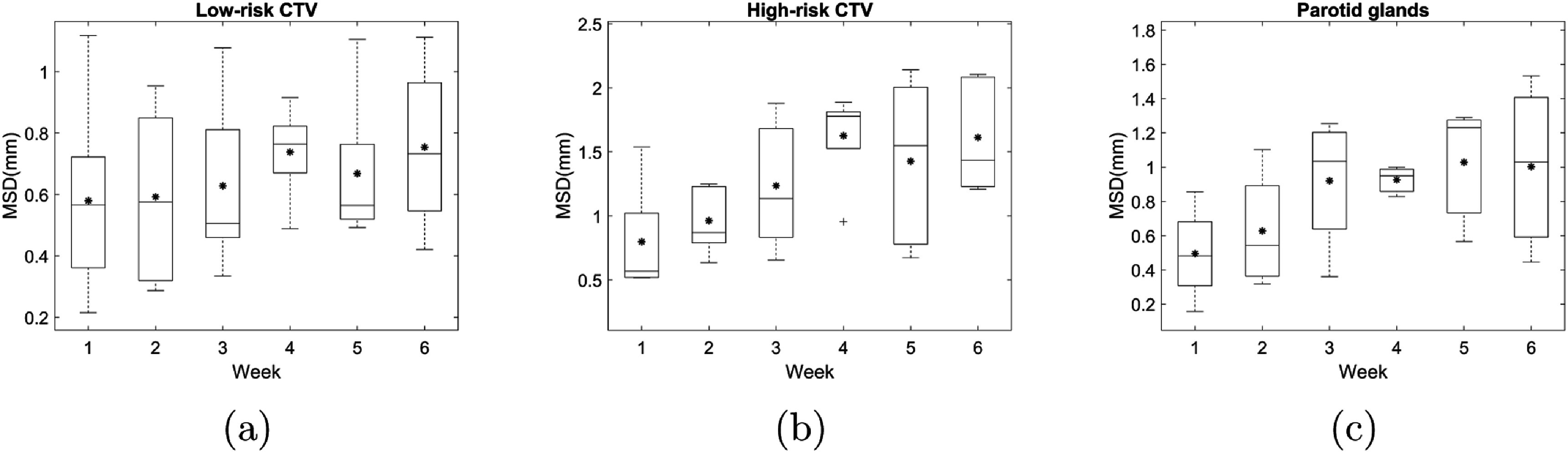
The weekly MSD between the deformed contours in dCTs and the corresponding contours in the planning CT for high-risk CTV, low-risk CTV and parotid glands. In the box plot, the horizontal lines indicate the median value, and the asterisks indicate the mean value.

The weekly MSD between the deformed contours in dCTs and the corresponding contours in the pCT across the 5 test patients are shown for high risk CTV, low risk CTV and parotid glands in figure 1. The maximum MSD of all these structures was below 3 mm.

**Figure 2.**
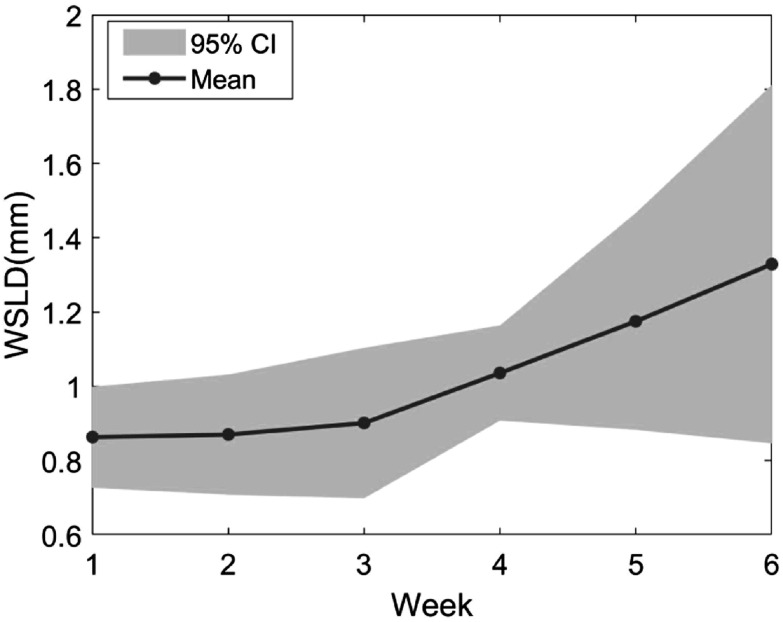
The weekly WSLD between dCTs and the corresponding pCT. The result is estimated in average WSLD with 95% CI over the 5 test cases.

The WSLD caused by the influence of the DIR algorithm in our test patients is shown in figure 2. In individual cases, minimum and maximum WSLD of 0.44 and 2.17 mm were found (< slice thickness of 3 mm). The average WSLD with 95% confidential interval (CI) across five test patients increased from 0.86 ± 0.14 mm (week 1) to 1.33 ± 0.48 mm (week 6). The weekly average was 1.03 ± 0.23 mm, which was close to the pixel size of 0.98 mm, showing the feasibility of the DIR for this study.

**Figure 3.**
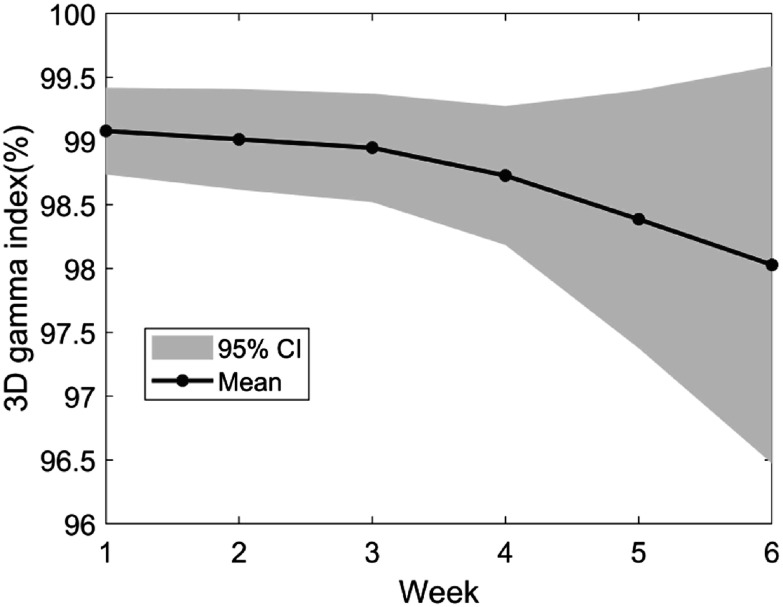
The weekly gamma index between the dose distribution on dCTs and the corresponding pCT. The result is estimated in average WSLD with 95% CI across the 5 test cases.

The weekly gamma index between the dose distribution on dCTs and the corresponding pCT is shown in figure 3 for 5 test cases. dCTs should have the same dose distribution as the pCT in an ideal DIR. The average gamma index was reduced from 99.05% (week 1) to 98.03% (week 6), but all the gamma index >95%, which is the standard passing rate generally accepted (Jin *et al* 2015, Szczurek *et al* 2019).

The above results justify the use of this DIR algorithm for anatomical models.

### 3.2. Anatomical model evaluation based on HU

**Figure 4.**
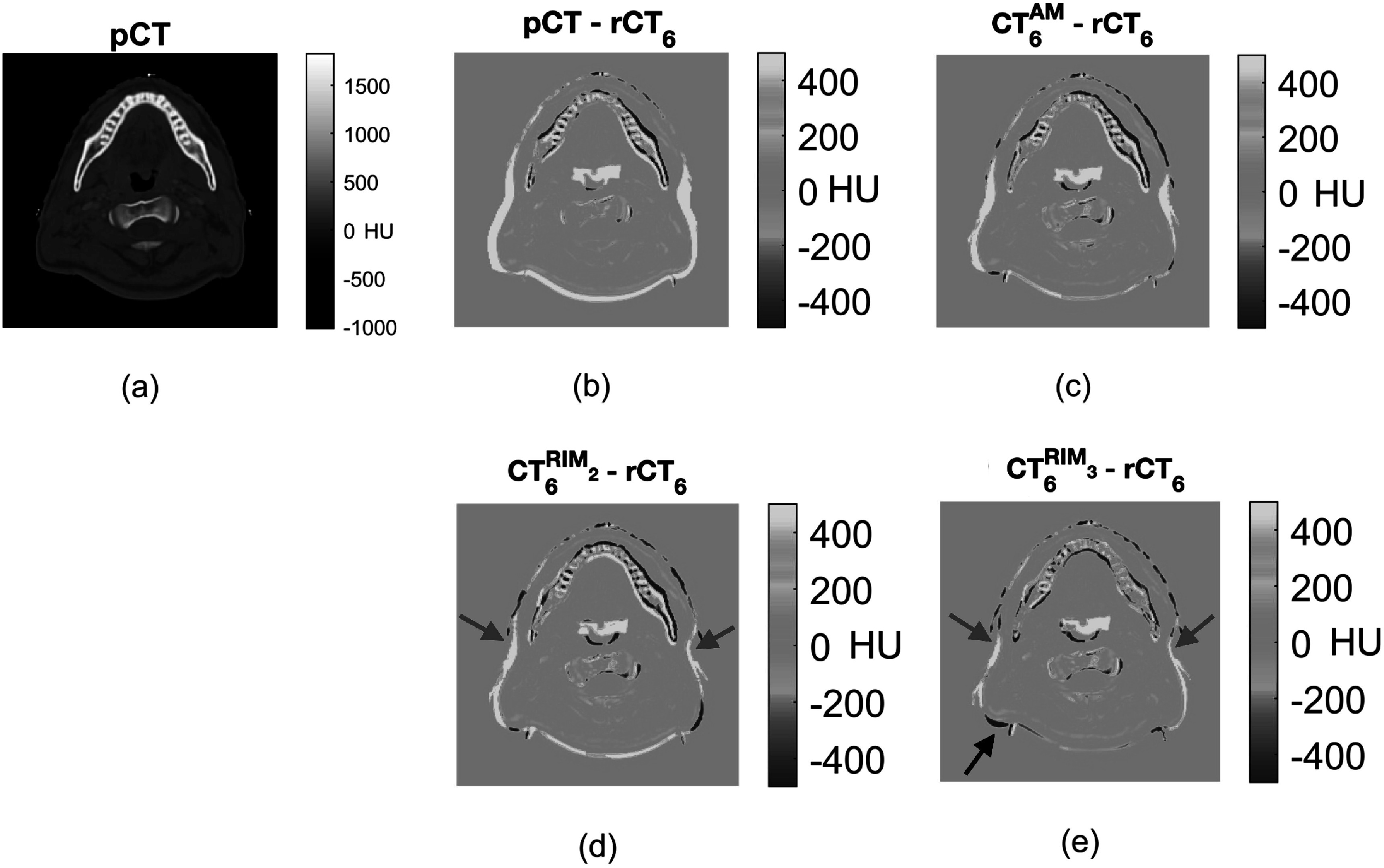
Comparison between different anatomical models using a representative example patient. (a) shows a slice from the pCT of a patient in the test dataset. (b) shows the difference image between pCT and rCT_6_, without the application of any anatomical model (no model). (c) is the difference image between predicted CT from the AM and rCT_6_. (d) is the difference image between predicted CT from the RIM_2_ model and rCT_6_. (e) is the difference image between predicted CT from the RIM_3_ model and rCT_6_.

In this section, we compared the image difference on HU between rCT_6_ and corresponding predicted CT_6_ from the 5 test patients. For visual assessment, figure 4 shows a slice of image differences of a test patient.

The shrinkage from pCT to rCT_6_ is indicated by the yellow area in figure (4 2(b)). This shrinkage leads to protons travelling further and causes a dose discrepancy as a result. From visual assessment, with the AM, the yellow area is reduced in 2(c)). The RIM predicted more accurately the anatomical changes of this patient in the area pointed by the red arrows. The refinement from the RIM_3_ further reduced this difference but overestimates the posterior shrinkage, indicated by the black arrow.

**Figure 5.**
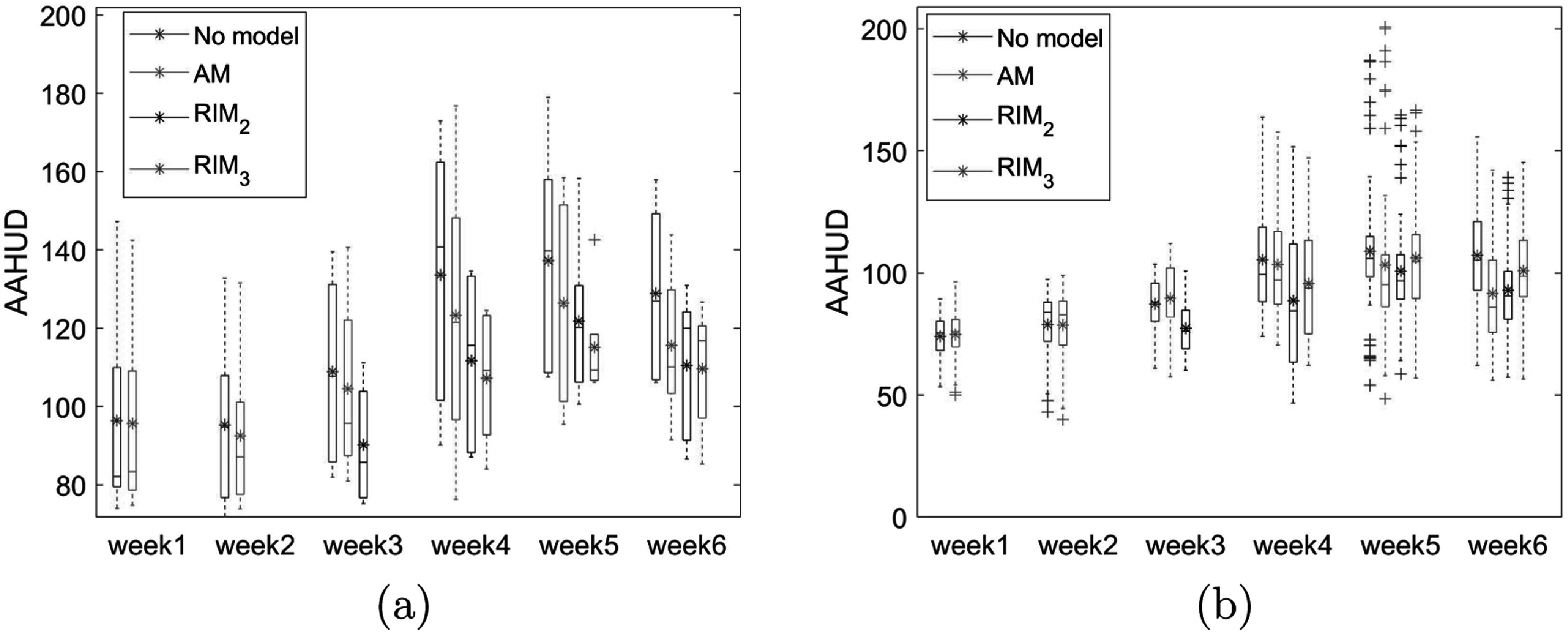
Boxplot of the AAHUD analysis: (a) shows the average AAHUD from the 5 test cases; (b) the AAHUD from a special case. The range shows the AAHUD of different image slices. The horizontal lines in the box plot indicate the median value, and the asterisks indicate the mean value.

The weekly AAHUD (no model, AM, RIM_2_, and RIM_3_) over all test patients with approximately 8 million voxels in total and a special case with approximately 2 million voxels are analysed and shown in figures 5(a) and (b), respectively. Because we used the deformation of one or two weeks to refine the model, the AAHUD of the RIM_2_ is shown from week 3 to week 6, and the AAHUD of the RIM_3_ is shown from week 4 to week 6.

In the special case (figure 5(b)), we observed no improvement from the RIM_3_ compared to the RIM_2_, with only small HU differences between the two models. On average, compared to no model, AM, RIM_2_, RIM_3_ reduced the AAHUD by 13.6 HU, 18.4 HU, 19.2 HU respectively at week 6. RIM_3_ captured more characteristics of the individual anatomical changes and had a higher predictive ability than the RIM_2_.

### 3.3. Model evaluation based on contours

**Figure 6.**
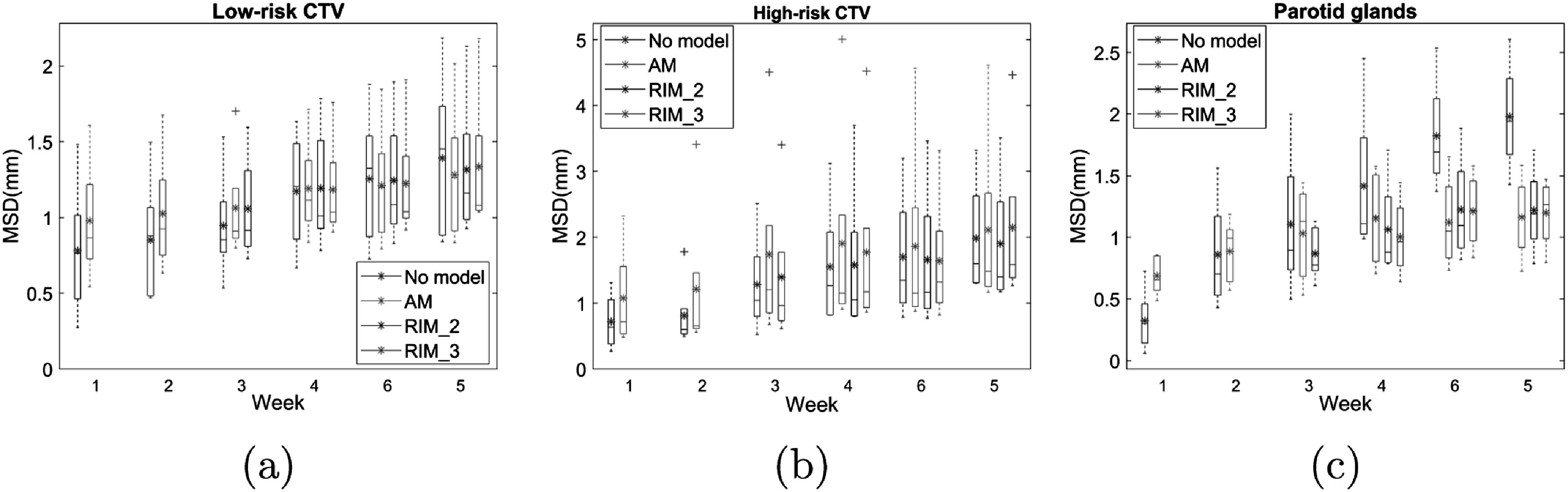
The weekly MSD between the predicted contours of the models and the corresponding contours in the rCT_*t*_ for high-risk CTV, low-risk CTV and parotid glands. In the box plot, the horizontal lines indicate the median value, and the asterisks indicate the mean value.

The weekly MSD between the predicted contours of the models and the corresponding contours in the rCT_*t*_ are shown for high risk CTV, low risk CTV and parotid glands in figure 6. When the predicted contours from the models were used, the average MSD of parotid glands can be reduced from 1.98 (no model) to 1.16 mm (AM) and 1.19 mm (RIM_3_) at week 6. No significant improvement was found on CTVs.

### 3.4. Model evaluation based on WSLD.

In this section, we estimated the range differences using the spot location of the treatment plans from 5 test patients.

**Figure 7.**
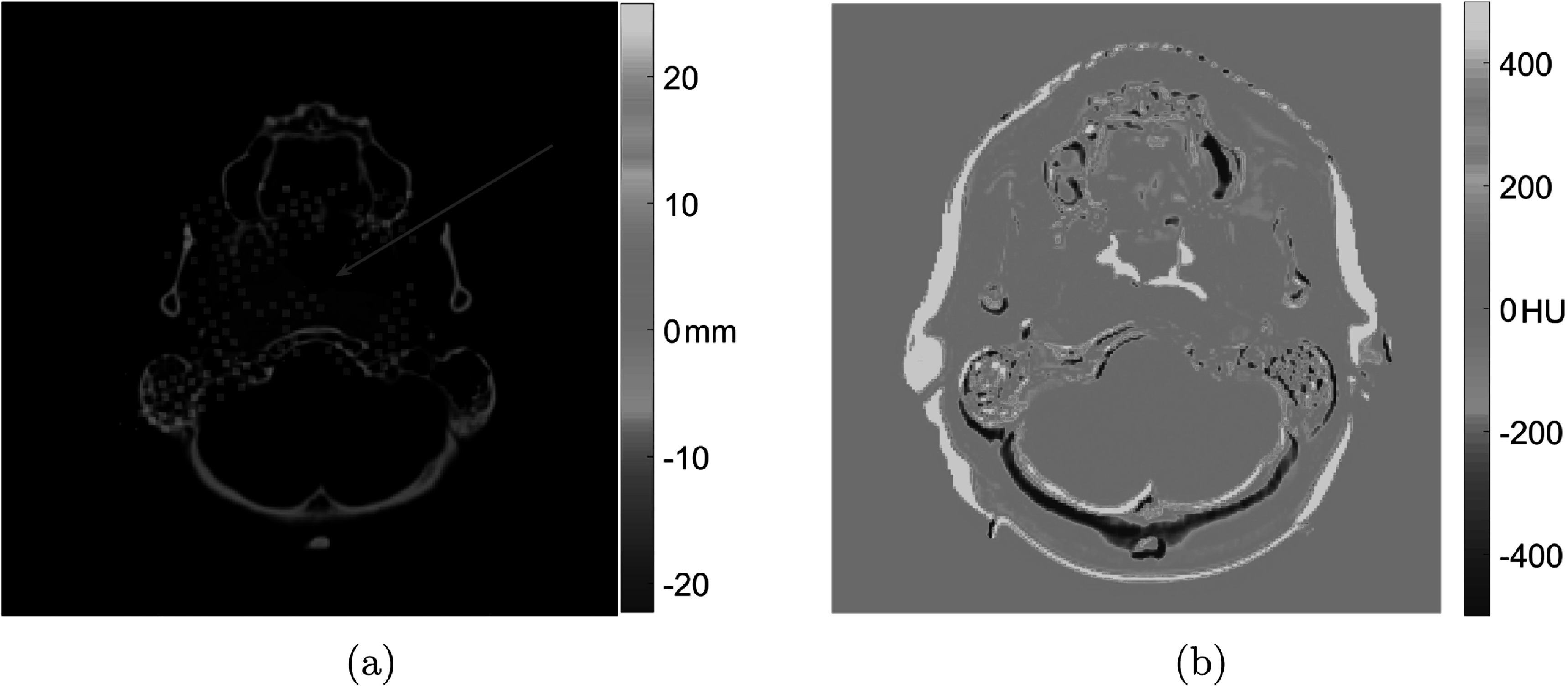
A slice of the spot error map between pCT and CT_6_. (a) is the spot error map of the beam angle 60°. The red area is the radiation target of beam angle 60°. Positive values mean spots go deeper along the beam path and negative values mean spots stop at shallower places. (b) is the image difference between pCT and CT_6_, as a reference for spot error map.

Range differences were represented by WSLD for each treatment beam of each test patient. For illustration, figure 7 shows an example of the treatment spots for one treatment field. In the figure, we show the proton spots that deviated from their original positions, with the magnitude of the deviation being colour coded as calculated from equation (13).

**Figure 8.**
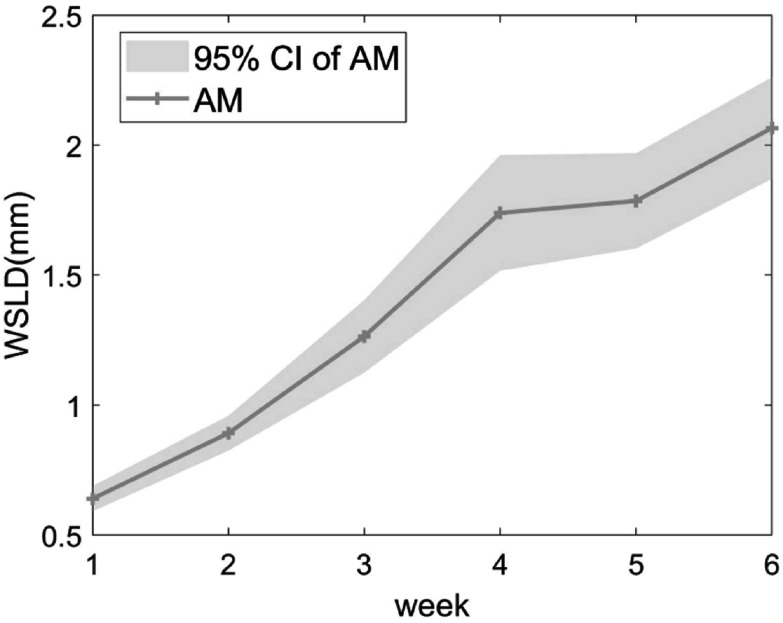
The systematic progression uncertainty estimated from the AM for each week. The result is estimated in average WSLD with 95% CI across the 5 test cases.

The WSLD originating from the systematic progression uncertainty estimated from the AM is shown in figure 8. The uncertainty from systematic progressions steadily increased to 2.07 ± 0.20 mm at week 6.

**Figure 9.**
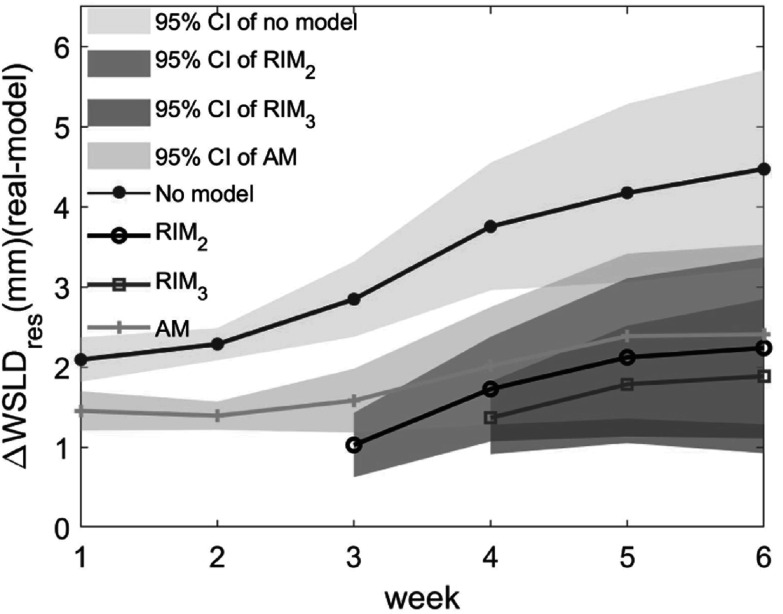
The residual anatomical uncertainty in WSLD. The residual anatomical uncertainty from the AM, the RIM_2_ and the RIM_3_ were compared. The graph shows the average difference with 95% CI between the estimated WSLD from the models and actual WSLD across the 5 test dataset.

The average WSLD from residual anatomical uncertainties from models and corresponding 95% CI were compared in figure 9. When the uncertainty estimated from the predicted images of the models was considered, the residual anatomical uncertainty was reduced from 4.47 ± 1.23 (no model) to 1.89 ± 0.96 mm (RIM_3_), 2.24 ± 1.13 mm (RIM_2_), 2.41 ± 1.12 mm (AM) at week 6, achieving significant improvements as compared to no model.

The comparison of individual cases between the four models (including no model) is listed in appendix D.

A summary of model uncertainty based on WSLD is listed in table 3.

**Table 3.** Summary of model evaluation based on WSLD over all test patients.

Week	Uncertainty (Mean ± 95%CI) (mm)				
	Model (Residual anatomical uncertainty)				DIR
	(No model)	AM	RIM_2_	RIM_3_	
1	2.09 ± 0.28	1.45 ± 0.24	—	—	0.79 ± 0.17
2	2.29 ± 0.20	1.39 ± 0.18	—	—	0.88 ± 0.20
3	2.85 ± 0.47	1.58 ± 0.40	1.03 ± 0.38	—	0.92 ± 0.22
4	3.75 ± 0.80	2.01 ± 0.73	1.73 ± 0.65	1.37 ± 0.45	0.99 ± 0.17
5	4.17 ± 1.11	2.39 ± 1.03	2.12 ± 0.99	1.79 ± 0.73	1.14 ± 0.29
6	4.47 ± 1.23	2.41 ± 1.12	2.24 ± 1.13	1.89 ± 0.96	1.33 ± 0.45

### 3.5. Model evaluation based on dose distribution.

**Figure 10.**
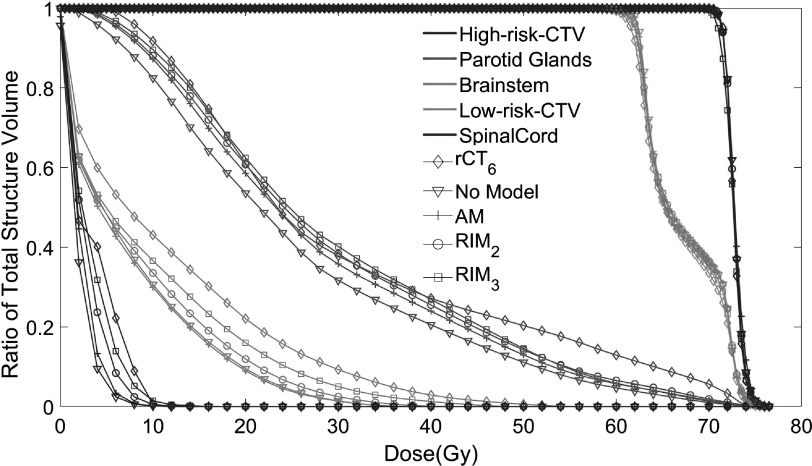
DVH curves for the dose distribution of a test patient (figure 4) at week 6 from the rCT_6_, the AM, the RIM_2_, the RIM_3_ and no model (planning CT).

Figure 10 shows the DVH curves for the dose distribution of a test patient (figure 4) at week 6 from the rCT_6_, the AM, the RIM_2_, the RIM_3_ and no model. We observed that the DVH of the RIM_3_ is the closest to the DVH of rCT_6_. The worst performance in the OARs is observed without using a model.

The results of the gamma analysis between the dose distribution on rCTs and the corresponding predicted weekly CTs from no model (predicted images were replaced by the pCT), the AM and the RIMs were listed for each patient in table 4. The number of cases of which gamma index < 95% was reduced from 9 (no model) to 6 (AM), to 4 (RIM_2_) and to 2 (RIM_3_). The average gamma index among 5 test patients was improved from 93.87 ± 2.48 % (no model) to 96.16 ± 1.84 % (RIM_3_) at week 6.

**Table 4.** The gamma index between the dose distribution on rCTs and the corresponding predicted weekly CTs from no model (predicted images were replaced by planning CT), the AM and the RIMs for each test patient and each week. Gamma indexes below 95%, the generally accepted standard passing rate, are highlighted in bold. The gamma indexes of week 1 and week 2 are only listed for no model and the AM as the RIM updated from week 3.

Id	week 1 (%)		week 3 (%)				week 5 (%)			
	No model	AM	No model	AM	RIM_2_		No model	AM	RIM_2_	RIM_3_
1	98.1	98.1	**93.2**	**94.1**	95.3		**91.2**	**93.4**	**94.6**	96.1
2	99.3	98.8	99.1	98.7	99.2		98.0	98.2	98.6	98.2
3	96.4	97.2	96.1	96.4	96.2		**91.4**	**91.8**	**91.6**	**91.5**
4	97.7	97.8	95.8	95.2	96.5		96.6	96.8	96.7	96.9
5	98.5	98.1	96.6	97.3	98.0		**93.4**	95.6	96.0	96.4
mean	98.01	98.01	96.16	96.33	97.06		**94.09**	95.16	95.50	95.81
CI	0.85	0.47	1.71	1.44	1.23		2.45	2.07	2.12	2.06
Id	week 2 (%)		week 4 (%)				week 6 (%)			
	No model	AM	No model	AM	RIM_2_	RIM_3_	No model	AM	RIM_2_	RIM_3_
1	95.3	95.6	**93.4**	95.5	96.6	97.5	**91.2**	**93.9**	**94.6**	97.5
2	99.3	99.2	98.2	97.9	98.4	98.4	97.3	97.9	98.2	97.3
3	95.0	96.7	95.5	96.9	94.8	96.0	**91.5**	**92.4**	**91.4**	**92.2**
4	97.3	97.0	95.7	94.8	96.3	96.6	97.1	96.7	97.5	97.7
5	98.2	98.2	**94.1**	96.7	97.5	97.3	**92.2**	**94.9**	95.6	96.0
mean	97.03	97.34	95.37	96.39	96.72	97.16	**93.87**	95.18	95.48	96.16
CI	1.49	1.14	1.47	0.98	1.09	0.72	2.48	1.76	2.15	1.84

## 4. Discussion

In this paper we developed and compared 2 different anatomical models. The AM is a basic model only used to evaluate systematic anatomical uncertainty. The RIM is a further refinement based on the AM, with the suggested use in offline adaptive treatment planning. The model accuracy was evaluated based on AAHUD, contours, WSLD and dose distributions.

### 4.1. Model evaluation

From the AAHUD comparison, on average, the RIM predicted the anatomical changes with the highest accuracy when compared with the AM or no model. This observed outcome was expected because the deformation differences include the progressive variation between patients. It is important to note that small random anatomical changes such as jaw movement and shoulder position changes will also be included in the deformation differences to update the model. If the magnitude of random variation was greater than the progressive variation, the RIM can be inferior to the AM, as shown in figure 5(b). The patient shown in this case was very slim at the start of treatment and had <5% weight loss. The random anatomical changes can be more predominant, making the RIM less effective. Nevertheless, the observed differences between the AM and the RIM were small.

The model evaluation based on contours showed that models are more effective in predicting the changes of parotid glands. Because we did not stratify patients based on CTV features, and CTV location and size are diverse in this dataset, predicting the changes of the CTVs is challenging. This contour-based model evaluation assumes that the contours were perfect on all CTs. In this study, the inter-observer variation was eliminated as a single physician contoured the organs and the intra-observer variability was minimized by the use of a copy-and-modification strategy (Tan *et al* 2013). In reality, intra-rater variability exists and can lead to an increase in the evaluation metrics.

The spot error map gave us an intuitive visual view of possible spot location variation, which can guide the use of beam angles and the design of the objectives in the optimisation. For example, the error map can capture the ‘dangerous spots’ with high variations, which might damage critical normal tissue. Therefore, we can avoid that spot position or increase the weight of normal tissue protection in the optimisation procedure.

As shown in figure 8, we found that the effect of anatomical progressions in the first week of treatment was not significant, justifying our approach to refine the model from week 2 onward. The RIM_3_ can reduce the anatomical uncertainty from 4.47 ± 1.23 mm (no model) to 1.89 ± 0.96 mm at week 6 (see figure 9).

In table 4, the average gamma indexes throughout the 5 test patients between the dose distribution on rCTs and the corresponding predicted weekly CTs from the AM and the RIM are all above 95% for each week, which is the standard passing rate generally accepted (Jin *et al* 2015, Szczurek *et al* 2019). Also, the average gamma index was improved from 95.18 ± 1.76% (AM) to 96.16 ± 1.84 (RIM_3_) at week 6. Combining with figures 5 and 9, we demonstrated that the RIMs can be gradually refined during the treatment and can potentially serve as a routine monitor to update the prediction and prepare adaptive intervention if necessary.

In comparing individual gamma values in table 4 and individual WSLD in table C3 in appendix C, there is a high level of consistency of 80% in terms of selecting the best prediction, thereby validating the feasibility of using WSLD as an evaluation tool.

### 4.2. Study limitations

For the purpose of validating our model, we used 20 patients with weekly CT imaging, which is used less frequently in routine clinics than cone-beam CT (CBCT), to reduce the error from HU corrections when calculating the spot location and dose distribution. The procedure of using CBCT images to build the model is the same except that the influence of DIR between CT and CBCT might be different and would need to be evaluated on an individual basis. The DIR between CT and CBCT has been investigated in the literature Zhen *et al* (2013), Veiga *et al* (2014).

The models were built based on a relatively small sample dataset of 15 patients, and analyses were performed on 5 patients. The weight loss of these 5 patients ranges from 4% to 18% (the weight loss of the training dataset ranges from 2% to 12%), including patients with small anatomical changes and severe anatomical changes. We also exploited the current dataset and measured the sensitivity of the AM towards the training data by repeatedly sampling random training data for the same patient in appendix E. The 95% CI of different measurements is less than 0.12 mm. However, it cannot completely remove the concerns of over-fitting when the model is directly applied to another dataset. We are currently working on finding the optimal parameters to build models for patients with CBCT data. It requires a relatively large dataset size to avoid the risk of over-fitting. Further validation of the model and the estimation of the sensitivity will be conducted on a larger cohort of patients. When a large dataset is available, patient stratification can also be used to improve the model accuracy. The model built based on a cohort of patients with the same characteristics can be applied to the same type of patients. The features that might be related to the anatomical changes have been revealed (Brivio *et al* 2018, Lassen *et al* 2009, van Dijk *et al* 2017, Gabryś *et al* 2018, Bogowicz *et al* 2019). Assuming the model is built based on a large dataset with delicate stratification, the accuracy of the model should be only limited by the DIR uncertainty and small non-rigid positioning uncertainties.

Another limitation of the presented study is that the patients used to build and evaluate the models have received photon therapy, and we assumed that patients undergoing proton therapy have similar anatomical changes as in photon therapy. Further validation of the model will be conducted on a cohort of patients treated with IMPT.

## 5. Conclusion

We have presented and analysed different anatomical models for H&N patients. The results demonstrated the ability of the models to predict anatomical changes during the course of radiotherapy. Additionally, the influence of individual and cumulative uncertainties on the position of the proton beam spots was investigated. The exploration of potential clinical applications, such as the use of anatomical models to prepare offline adaptive plans, is underway.

## Figures and Tables

**Figure B1.**
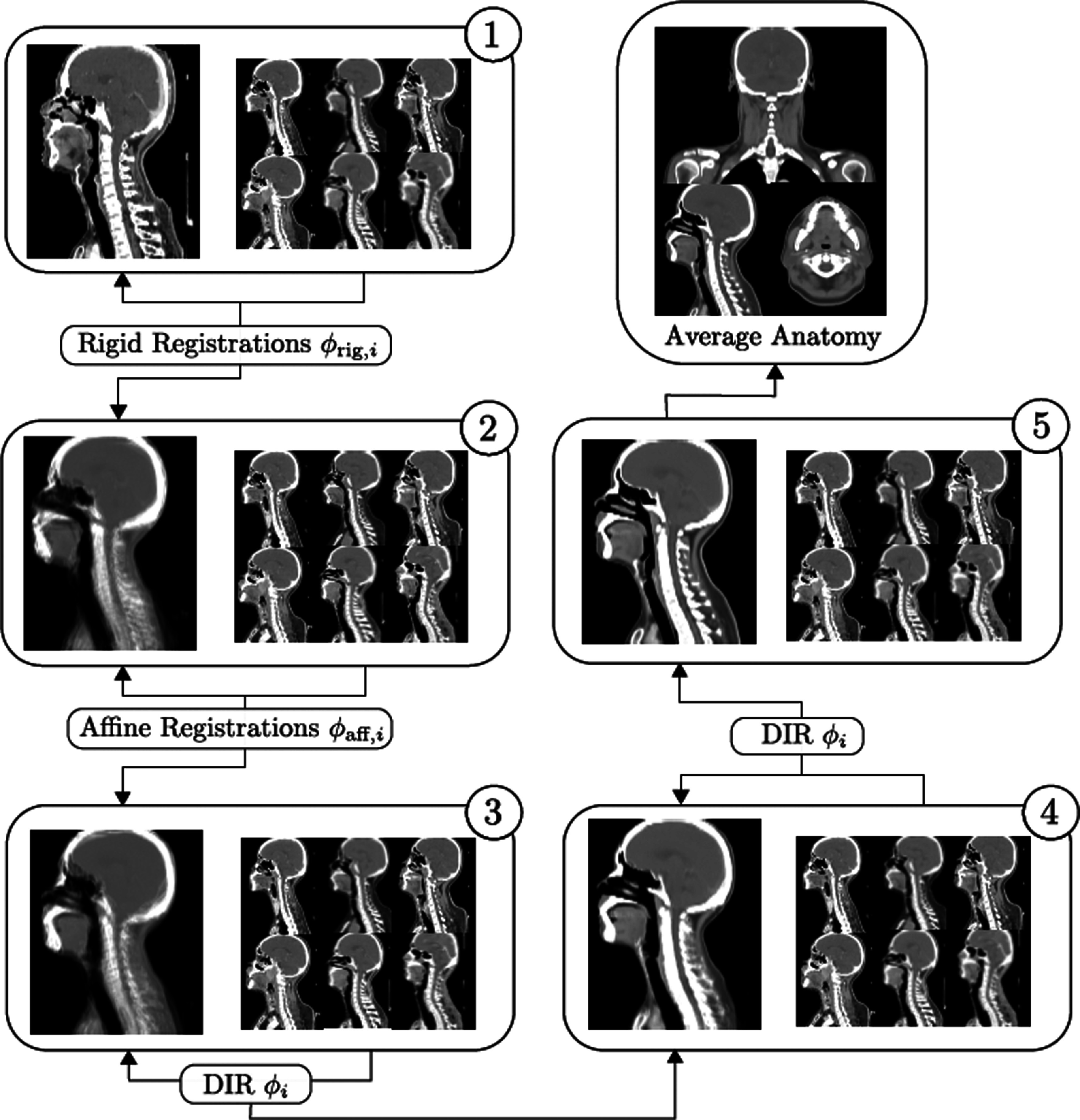
Group-wise registration building the reference anatomy. The average image represents the mean shape of the cohort of patients used to perform the group-wise registration.

**Figure E1.**
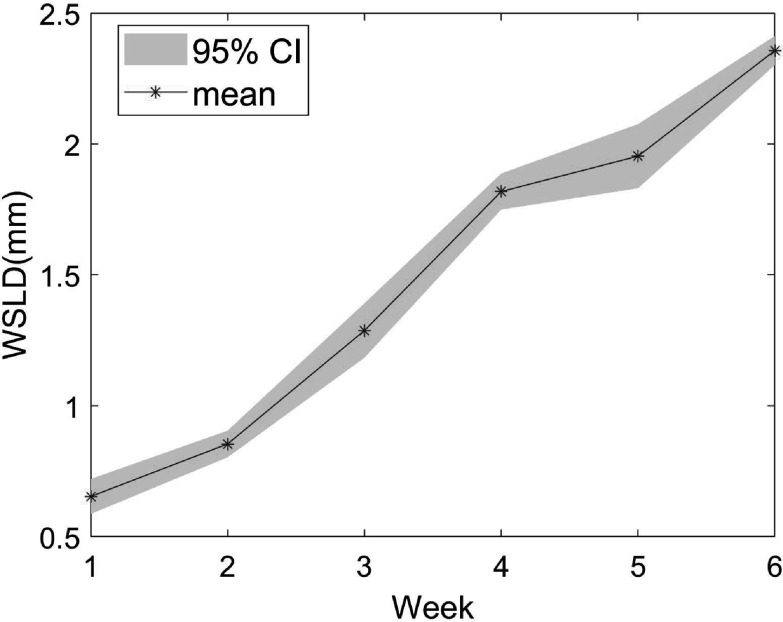
The measurement of model sensitivity for the AM. The WSLD of the AM was calculated 5 times based on 5 groups of training data, referred to as WSLD${}_{\mathrm{AM}}^{\mathrm{sensitivity}}$. The figure shows the mean value and 95% CI of the WSLD${}_{\mathrm{AM}}^{\mathrm{sensitivity}}$.

## Funding statement

Ying Zhang is supported by the scholarship from China Scholarship Council Nos. 201 809 150 003. Esther Bär is supported by the Radiation Research Unit at the Cancer Research UK City of London Centre Award C7893/A28990. Wenyong Tan is supported by the National Natural Science Funding of China (81974462).

## Conflict of interest

None

## Data share statement

Restricted access. The authors cannot make patient data publicly available due to data use agreement. The programming code of building anatomical models are available on the GitHub https://github.com/YingClara92/Anatomical_Models.git.

## Appendix A. Patient image acquisition details

CT images of these 20 patients were all acquired using a Brilliance Big Bore CT simulator (Philips, Inc, Cleveland, OH, USA). The details are listed in table A1.

**Table A1.** CT image acquisition details.

Tube voltage	Reconstruction diameter	Slince thickness	Pixel spacing	Data collection diameter
120 kVp	500 mm	3 mm	0.98 mm	600 mm

## Appendix B. Generation of an average reference anatomy for statistical models

A group-wise registration algorithm was adapted from the NiftyReg package to generate an ‘average’ atlas. Group-wise registration can be used to spatially normalise a cohort of patients in a common space. Using *N* different patient CT images, the iterative algorithm consists of the following 6 steps:

(i) perform rigid registration between *N*-1 other CT *I*_*i*_ and an arbitrary reference image *I* ^*^. The warped image ${I}_{i}^{{\prime} }={I}_{i}({{\mathrm{T}}}_{\mathrm{rigid}},i)$. The template image is updated as the average of all N images:
  \begin{eqnarray*}{I}^{* }=\displaystyle \frac{1}{N}\left({I}^{* }+\sum _{i=1}^{N-1}{I}_{i}^{{\prime} }\right)\hspace{2cm}{I}_{i}^{{\prime} }={I}_{i}({{\mathrm{T}}}_{\mathrm{rigid}}).\end{eqnarray*}
(ii) Perform affine registration. All *N* images are registered to *I* ^*^, producing the affine transformations *ϕ*_aff,*i*_ for each iteration.
(iii) Update *I* ^*^ with a series of affine iterations. To enforce the mean of all the transformations to be the identity, the mean of the non-rigid components of the affine transformations *ϕ*_aff,*i*_ is removed using ${\phi }_{\mathrm{aff},i}^{{\prime} }={\phi }_{\mathrm{aff},i}-\tfrac{1}{N}{\sum }_{i}{\phi }_{\mathrm{aff},i}$. *I* ^*^ is updated using the average of the ${\phi }_{\mathrm{aff},i}^{{\prime} }$:
  \begin{eqnarray*}{I}^{* }=\displaystyle \frac{1}{N}\sum _{i=1}^{N}{\phi }_{\mathrm{aff},i}^{{\prime} }({I}_{i}).\end{eqnarray*}
(iv) Steps (ii) and (iii) are repeated until no visual improvement of quality in *I* ^*^.
(v) Perform diffeomorphic DIR. All *N* images *I*_*i*_ are deformed to the current template image *I* ^*^ using the stationary velocity field *v*_*i*_.
(vi) Update *I* ^*^ with a series of DIR iterations. The spacing of the control points for the B-Spline velocity grid is gradually stepped down from coarse to fine (30–8 mm) during the iteration. For each iteration, remove the mean of the velocity field from each transformation ${v}^{{\prime} }=v-\tfrac{1}{N}{\sum }_{i}{v}_{i}$ as before. The average image is computed as:
  \begin{eqnarray*}{I}^{* }=\exp \left(\displaystyle \frac{1}{N}\sum _{i=1}^{N}{v}^{{\prime} }({I}_{i})\right).\end{eqnarray*}

The illustration of group-wise registration is shown in figure B1.

## Appendix C. Acquiring spot location

For each of the 5 test patients, the treatment plans were exported from the Varian treatment planning system. Information of the spot positions (*X*, *Y*) and energy/layer (*Z*) was extracted from the plan files. (*X*, *Y*) are recorded relative to the isocentre (the centre of the target). (*X*, *Y*) with the beam angle can specify the beam’s path. The beam energy (*Z*) determines the depth of the spot along the path by calculating the WEPL using equation (C.1).

\begin{eqnarray*}{WEPL}=\sum _{i,j,k\in S}{{RSP}}_{i,j,k}\cdot {d}_{i,j,k},\end{eqnarray*}

where *S* is a set of voxels that contain the beam path. RSP_*i*,*j*,*k*_ is the voxel-wise relative stopping power estimated from CT numbers using a clinical calibration curve. *d*_*i*,*j*,*k*_ is the path length of the beam inside voxels (*i*, *j*, *k*), estimated by a ray-tracing algorithm (Lui 2018). The beam-lines are assumed parallel in this study.

## Appendix D. Individual cases

The WSLD for each model in each test patient and week is listed in table D1. The bold text marks the closest WSLD number estimated from models to the real WSLD number from rCT.

**Table D1.** WSLD for each model in each test patient and week. The numbers of the models that are the closest to the corresponding number of rCT are highlighted in bold as the best prediction.

**ID**	**rCT(mm)**	**AM(mm)**	**RIM** _**2(mm)**	**RIM** _**3(mm)**
id = 1				
week1	1.72	0.57	—	—
week2	2.52	0.84	—	—
week3	3.43	1.18	**1.78**	—
week4	4.93	1.65	2.11	**2.92**
week5	5.62	1.70	2.18	**2.95**
week6	5.23	2.00	2.53	**3.25**
id = 2				
week1	1.96	0.60	—	—
week2	2.07	0.80	—	—
week3	2.15	1.06	**1.77**	—
week4	2.78	1.34	**2.00**	1.91
week5	2.73	1.47	**1.98**	1.91
week6	3.12	1.71	2.11	**2.15**
id = 3				
week1	2.57	0.66	—	—
week2	1.99	0.86	—	—
week3	2.59	1.32	**1.56**	—
week4	2.94	**1.91**	1.70	2.22
week5	4.02	1.79	1.56	**2.17**
week6	5.13	2.12	1.75	**2.24**
id = 4				
week1	2.32	0.73	—	—
week2	2.30	0.96	—	—
week3	2.57	1.23	**1.85**	—
week4	3.44	1.73	1.90	**2.00**
week5	2.91	1.87	2.01	**2.04**
week6	2.62	2.12	2.13	**2.22**
id = 5				
week1	1.89	0.65	—	—
week2	2.54	1.00	—	—
week3	3.49	1.53	**2.12**	—
week4	4.69	2.07	2.43	**2.89**
week5	5.57	2.10	2.49	**2.88**
week6	6.27	2.38	2.68	**3.07**

## Appendix E. Sensitivity of model

Because the AM is the basic model, the sensitivity measurement was conducted on the AM using WSLD. To measure the sensitivity of the AM to the training data, we randomly selected one test patient. Then, we randomly selected 5 × 15 patients from the remaining 19 patients as training set, resulting in 5 groups of sensitivity training data for the test patient. For each sensitivity training dataset, the WSLD in the test patient was calculated (WSLD${}_{\mathrm{AM}}^{\mathrm{sensitivity}}$). The 95% CI of WSLD${}_{\mathrm{AM}}^{\mathrm{sensitivity}}$ is used as a measure for the sensitivity to the training data. The result shows only small differences between the 5 groups, see figure E1. Because there is a 15/19 chance that the sensitivity training data include the data used in the original training dataset, this measure only represents the sensitivity of the model based on this cohort of patients. Another group of patient data is required to fully measure the sensitivity of the model to the small set of training data.
